# Current and Future Implementation of Digitally Delivered Psychotherapies: An Exploratory Mixed-Methods Investigation of Client, Clinician, and Community Partner Perspectives

**DOI:** 10.3390/healthcare12191971

**Published:** 2024-10-03

**Authors:** Sidney Yap, Rashell R. Allen, Carley R. Aquin, Katherine S. Bright, Matthew R. G. Brown, Lisa Burback, Olga Winkler, Chelsea Jones, Jake Hayward, Kristopher Wells, Eric Vermetten, Andrew J. Greenshaw, Suzette Bremault-Phillips

**Affiliations:** 1 Department of Psychiatry, Faculty of Medicine and Dentistry, University of Alberta, Edmonton, AB T6G 2H5, Canada; syap@ualberta.ca (S.Y.); burback@ualberta.ca (L.B.); ow@ualberta.ca (O.W.); andy.greenshaw@ualberta.ca (A.J.G.); 2 Heroes in Mind, Advocacy, and Research Consortium, Faculty of Rehabilitation Medicine, University of Alberta, Edmonton, AB T6G 2G4, Canada; caquin@ualberta.ca (C.R.A.); kbright@ualberta.ca (K.S.B.); mbrown2@ualberta.ca (M.R.G.B.); cweiman@ualberta.ca (C.J.); 3 School of Clinical Child Psychology, Faculty of Education, University of Alberta, Edmonton, AB T6G 2G5, Canada; wozniak@ualberta.ca; 4 Department of Occupational Therapy, Faculty of Rehabilitation Medicine, University of Alberta, Edmonton, AB T6G 2G4, Canada; 5 School of Nursing and Midwifery, Faculty of Health, Community and Education, Mount Royal University, Calgary, AB T3E 6K6, Canada; 6 Department of Computing Science, Faculty of Science, University of Alberta, Edmonton, AB T6G 2E8, Canada; 7 Department of Emergency Medicine, Faculty of Medicine and Dentistry, University of Alberta, Edmonton, AB T6G 2T4, Canada; jhayward@ualberta.ca; 8 Department of Child and Youth Care, Faculty of Health and Community Studies, MacEwan University, Edmonton, AB T5J 4S2, Canada; wellsk25@macewan.ca; 9 Department of Psychiatry, Leiden University Medical Centre, P.O. Box 9500, 2333 ZA Leiden, The Netherlands; e.vermetten@lumc.nl

**Keywords:** implementation, implementation science, digital mental health interventions, digital delivery, psychotherapy

## Abstract

Introduction: Following the initial outbreak of the COVID-19 pandemic, mental health clinicians rapidly shifted service delivery from in-person to digital. This pivot was instrumental in maintaining continuity of care and meeting increased mental health service demands. Many mental health services have continued to be offered via digital delivery. The long-term implications of delivering services via digital media remain unclear and need to be addressed. Objectives: This study aimed to identify current micro (i.e., clinician–patient interactions), meso (i.e., clinician–clinic manager interactions), and macro (i.e., government–policy maker interactions) level issues surrounding the use of digital mental health interventions (DMHI). Such integrated assessments are important for optimizing services to improve treatment outcomes and client satisfaction. Methods: Participants were recruited between January 2022 and April 2023. Quantitative data were collected using a survey informed by the Hexagon Tool. Qualitative data were collected from online semi-structured interviews and focus groups and analyzed using rapid thematic analysis. Results: Survey data were collected from 11 client and 11 clinician participants. Twenty-six community partner participants were interviewed for this study. Client and clinician participants expressed satisfaction with the implementation of DMHI. Community partner participants generally agreed, reporting that such services will play an integral role in mental healthcare moving forward. Community partners shared that certain issues, such as uncertainty surrounding policies and regulations related to digital delivery, must be addressed in the future. Conclusions: Participants in this study supported the use of DMHI despite difficulties implementing these programs, asserting that such services are not a temporary fix but a pivotal cornerstone in the future of mental healthcare service delivery.

## 1. Introduction

Mental healthcare service delivery underwent a substantial transformation in the wake of the COVID-19 pandemic. An escalation in mental health concerns stemming from the pandemic and a need to adhere to physical distancing mandates and social restrictions [1] necessitated the rapid and unprecedented implementation of digital mental health interventions ((DMHI) e.g., web conferencing platforms or telephone-based services) [2]. The pandemic was an unanticipated catalyst and contextual factor for innovation and system change that played a critical role in implementing novel programs [3]. The paradigm shift towards digital delivery that arose during COVID-19 offers many lessons that can inform future system change related to the implementation of DMHI.

The use of digitally delivered mental health services was significantly different prior to, during, and following the pandemic. Before the pandemic, DMHI use was limited to a narrow range of settings and applications [4], such as the provision of Cognitive Behavioral Therapy (CBT) by a mental health clinician synchronously working with an individual or group over video web conferencing [5]. As healthcare systems adjusted their practices to meet the treatment needs of patients during the pandemic while complying with public health mandates [6], many psychotherapeutic modalities were successfully adapted to digital delivery, including Cognitive Processing Therapy (CPT) [7], Eye Movement Desensitization and Reprocessing (EMDR) [8], and Prolonged Exposure (PE) [9]. Digital mental health supports came to be increasingly used by Canadians to access health information and coordinate and receive virtual care [10].

Research promisingly shows that digitally delivered services, such as internet-based CBT [11], appear to provide similar treatment effectiveness to in-person programs [12,13]. A number of unique advantages may be attributed to digital delivery, including increased access to treatment [14,15] and client cost and time savings [6]. Using DMHI may also reduce stigma experienced by clients when accessing mental healthcare [16]. Those who have difficulties accessing in-person services (e.g., child-caring parents, individuals living in rural communities, transportation issues, etc.) are among the many individuals who may benefit from the spread of DMHI. There are certain limitations to DMHI, however, including a need for baseline technological literacy from patients and therapists, additional training to ensure therapists feel comfortable providing digital care, and specialized clinical processes [15]. As the evidence supporting the use of DMHI increases [17], so too does the potential for their broad implementations.

With the height of the pandemic behind us, an opportunity has arisen to systematically consider the strengths and weaknesses of DMHI and evaluate their short- and long-term viability within the healthcare system. Implementation science provides a helpful paradigm for such investigations. The field aims to “promote the systematic uptake of research findings and other evidence-based practice into routine practice and, hence, to improve the quality and effectiveness of health services.” [18] (p. 1). Being intentional, explicit, and systematic around key issues and factors affecting program implementation [19] can better 1. inform spread (i.e., the adoption and replication of programs within a health system) and scale (i.e., system/infrastructure that impacts the full-scale implementation of programs) [20]; 2. facilitate translation into practice [21,22]; and 3. accelerate the adoption of interventions more quickly than the typical 17–20 years [21,22] it tends to take to do so. The population at large stands to greatly benefit from the timely, effective, and efficient implementation of DMHIs.

The aim of this study was to investigate and identify factors that may affect the current and future implementation of DMHI in mental healthcare systems. Diverse perspectives were integral to this exploratory study, including those of clients who received DMHI, mental health clinicians who provided DMHI, and community partners (i.e., individuals with connections to DMHI, including mental health clinicians with managerial duties, individuals in leadership positions, researchers, and technology experts). We sought to identify current micro (clinician–patient interactions), meso (clinician–clinic manager interactions), and macro (government–policy maker interactions) level [23] factors related to the use of digitally delivered mental health services. This analysis was conducted as it may aid in optimizing services to improve treatment outcomes for clients and clinician satisfaction when providing these interventions.

## 2. Materials and Methods

This study was an exploratory mixed-methods study designed to be conducted in a community-engaged research setting [24,25]. Data were collected through a survey utilizing indicators from the Hexagon Tool [26], semi-structured interviews, and focus groups. Ethics approval was obtained from the University of Alberta’s Health Research Ethics Board (Pro00109065). Written informed consent was obtained from all study participants prior to engaging in research activities.

Clinician participants completed the survey over Research Electronic Data Capture (REDCap 14.5.2) [27,28,29], a secure web application used for building, managing, and completing online surveys and databases. Client and clinician participants completed the Perspectives on Economic Impact survey over REDCap. Participants from the community partner group were invited to participate in a 30- to 60-min semi-structured interview or focus group, conducted and recorded over Zoom (version 5.15), to explore their perspectives on the current implementation and future use of DMHI. Client and clinician participants were interviewed in a previous study [30].

### 2.1. Participant Inclusion Criteria

#### 2.1.1. Client Participants

Currently serving military members, veterans, and public safety personnel (PSP) who were receiving or who had received digitally delivered trauma therapy from a mental health clinician in Canada, either through an Operational Stress Injury Clinic (military member, veteran, and Royal Canadian Mounted Police clients) or private provider, were recruited. All client participants had a current or prior primary diagnosis of posttraumatic stress disorder (PTSD) and/or a trauma-related mental health disorder, which may have stemmed from operational injuries or past traumatic event(s) (e.g., adverse childhood events).

#### 2.1.2. Mental Health Clinician Participants

Clinician participants encompassed multidisciplinary mental health clinicians and providers practicing in Canada who provided digitally delivered psychotherapy to civilian populations and trauma-affected populations, composed of Canadian military members, veterans, and PSP. This included, but was not limited to, psychiatrists, psychologists, social workers, and nurse practitioners who are able to provide psychotherapy in Canada in accordance with their provincial regulatory bodies.

#### 2.1.3. Community Partner Participants

Participants included mental health clinicians with managerial duties who actively provided mental health services, individuals within leadership positions, researchers, and technology experts. All participants were users or experts in the field of DMHI and had used DMHI throughout the course of the COVID-19 pandemic.

### 2.2. Participant Exclusion Criteria

Individuals who were under 18 years of age and/or unable to provide informed written consent and/or not fluent in English were excluded from participation in all study groups.

### 2.3. Recruitment and Data Collection

Participants were recruited between January 2022 and April 2023 from various partner and non-partner mental health clinics, research groups, government agencies, and mental health advocacy groups. Client and clinician participants were recruited from within Canada. Partner participants were originally only recruited from within Canada, with international recruitment starting following study commencement to attempt to increase participant numbers.

Potential participants must have had experience using digital mental health tools, including Zoom and computer-based therapy applications (e.g., apps used for CPT or EMDR). A number of recruitment strategies were used (i.e., word of mouth, snowball, and purposeful sampling). Interested participants who completed a consent to contact form over REDCap were contacted by a member of the research team by telephone or email to discuss the study, determine eligibility, and assess their willingness to participate voluntarily.

A link to a REDCap webpage was shared with eligible individuals to complete the study participant informed consent form. Participants completed survey measures or a semi-structured interview or focus group according to the study group. The survey and interview script used in this study were iteratively developed by the research team to ensure the integration of principles of equity, diversity, and inclusion, minimizing study burden, and maximizing data quality, collection, and analyses. Data collection took place from February 2022 to May 2023. Study data were stored on the REDCap server and a dedicated, encrypted, and password-protected research drive hosted by the Faculty of Rehabilitation Medicine at the University of Alberta. 

### 2.4. Demographics

#### 2.4.1. Client Participants

Eleven Canadian military members, veterans, and PSP completed survey measures. Client participants had an average age of 50 ± 10.5 years (range: 34 to 61 years old) and self-identified as female (*n* = 3), male (*n* = 8), White (*n* = 9), and Aboriginal/Metis (*n* = 2). All participants identified as heterosexual, and no participants identified as transgender or gender diverse. Our participants self-reported being in the CAF (*n* = 7), being in the police force (*n* = 2), as a paramedic (*n* = 1), or as a correctional worker (*n* = 1), with an average length of service of 21 ± 9.6 years (range: from 5 to 38 years). Four participants had received digitally delivered psychotherapies prior to engaging in this study, while seven were receiving digitally delivered psychotherapies for the first time during study participation. Participants did not disclose whether the psychotherapies they had or were currently receiving were TFP. All participants had received psychotherapy via video conferencing, with some reporting having received additional services via telephone (*n* = 3).

#### 2.4.2. Mental Health Clinician Participants

Eleven Canadian mental health clinicians completed survey measures. Clinician participants self-identified as female (*n* = 8), male (*n* = 3), and white (*n* = 11), with an average age of 46 ± 8.6 years (range: 33 to 58 years). All participants identified as heterosexual, and no participants identified as transgender or gender diverse. Clinician participants held a variety of clinical roles, including social worker (*n* = 4), registered psychologist (*n* = 3), psychiatrist (*n* = 2), and mental health therapist (*n* = 2). Workplaces were highly varied and included working at a community mental health clinic (*n* = 4), the provincial health authority (*n* = 3), private practice (*n* = 3), or a regional health service (*n* = 1). Several participating clinicians (*n* = 6) had not provided any form of digital delivery prior to the COVID-19 pandemic. At the time of study participation, six clinicians exclusively provided digitally delivered care, while the remaining five clinicians provided a combination of digitally delivered and in-person care.

#### 2.4.3. Community Partner Participants

Twenty-six community partners took part in a semi-structured interview or focus group. Seventeen community partners participated in a semi-structured interview (9 females and 8 males), while the remaining nine took part in one of four focus groups (7 females and 2 males). All participants identified as heterosexual, and no participants identified as transgender or gender diverse. Community partners were from a number of countries, including Canada (*n* = 22), Australia (*n* = 2), the Netherlands (*n* = 1), and Ukraine (*n* = 1). Participants included mental health clinicians with managerial roles (*n* = 10), leaders (*n* = 6) (e.g., senior leadership within Veterans Affairs Canada, senior clinic manager with Alberta Health Services), researchers (*n* = 8), and technology experts (e.g., information technology personnel) (*n* = 2).

### 2.5. Tools and Measures

#### 2.5.1. Readiness Survey: Digital Delivery of Trauma Therapy

Clinician participants retrospectively filled out the readiness survey as it pertained to each of three timepoints (pre-COVID-19, early COVID-19, and post-COVID-19, as described below), in a single data collection session. The Hexagon Tool is an implementation science tool used to assess the potential suitability of innovations within an implementation site. Specifically, the tool can be used to better understand how a new or existing program or practice fits into an implementing site’s existing work and context [26]. It can also be used as a planning tool to guide selection and assess the fit and feasibility of potential programs and practices for use and to better understand the readiness to implement a program. The Hexagon Tool assesses the fit and feasibility of implementing evidence-based innovations for general clinical purposes through six indicators: need, supports, capacity, usability, evidence, and fit [26].

For the purposes of this study, a seven-item questionnaire exploring implementation, need, evidence, fit, usability, capacity to implement, and supports related to digitally delivered psychotherapies was developed informed by the above six indicators and previous literature (see Appendix A for a copy of the readiness survey informed by the Hexagon Tool; exploration of specific psychotherapeutic interventions (e.g., CBT and PE), applications used for digital delivery of psychotherapies (e.g., web-based applications used to digitally deliver EMDR), or specific policies related to psychotherapy delivery using the survey tools was beyond the scope of this study). Clinician participants were asked to rate their level of agreement with each item in relation to three separate timepoints: (1) pre-COVID-19; (2) early COVID-19; and (3) post-COVID-19 (after the transition to digital delivery). Agreement ratings for the “before COVID-19” and “during the transition to digital delivery” timepoints were provided retroactively. The level of agreement was rated based on a seven-point Likert scale from 1 (strongly disagree) to 7 (strongly agree). This conversion allowed participants to rate the implementation of digitally delivered psychotherapies informed by Hexagon Tool indicators across the course of the COVID-19 pandemic.

#### 2.5.2. Perspectives on Economic Impact

Client and clinician participants were asked two yes/no questions regarding their experiences using DMHI: did they save (1) money attending digitally delivered sessions; and (2) time attending digitally delivered sessions? If participants answered affirmatively, they provided an estimate of the money and time they saved. This cursory economic impact analysis was conducted to gain preliminary insight into the accessibility and efficiency of digitally delivered services.

#### 2.5.3. Interviews and Focus Groups

Community partner perspectives regarding the current implementation and future use cases of DMHI were captured through 30 to 60 min semi-structured interviews (*n* = 17) and focus groups (*n* = 4 total focus groups and *n* = 9 total participants), conducted and recorded over Zoom [31]. Key discussion topics included the previous and current state of implementation of DMHI; barriers to, facilitators of, and recommendations for the use of DMHI; and perspectives on how to optimize DMHI to client, clinician, and healthcare system needs. Interviews and focus groups were conducted until sufficient information power was reached [32].

See Appendix B for the interview scripts used for community partner interviews and focus groups.

### 2.6. Quantitative Data Analysis

All data were de-identified following data collection and analyzed using IBM SPSS Statistics software (Version 28.0) [33]. Descriptive statistics were calculated for each of the seven readiness survey items and two Perspectives on Economic Impact survey items.

Non-parametric analyses were conducted to analyze clinician participants’ readiness survey data due to the relatively small sample size. Friedman’s test was used to assess differences in readiness survey indicator scores across timepoints for each of the seven readiness survey items. The Wilcoxon signed rank test was then used for post hoc comparisons of indicator scores between pairs of timepoints: timepoints 1 and 2, 2 and 3, and 1 and 3 (timepoints described above). The Benjamini–Hochberg procedure was used to control the False Discovery Rate (FDR) to address multiple comparisons across the 21 Wilcoxon tests. 

### 2.7. Qualitative Data Analysis

Video-recorded interviews and focus groups were locally transcribed using Adobe Premiere Pro, with transcription accuracy verified by a research team member (S.Y.). Transcripts underwent rapid descriptive analysis following Gale et al. [34]. Transcript consolidation and summarization were conducted by three research team members (S.Y., C.R.A., and K.S.B.), and relevant information was extracted and thematically analyzed both deductively and inductively following an iterative process [35]. Initial codes were deductively developed based on interview and focus group topics and study objectives. Inductive coding involved identifying themes that emerged from collected data. A senior researcher (S.B.-P.) subsequently reviewed and refined the codes, which were then combined and tabulated into preliminary themes. Analysis of preliminary themes by the larger research team followed, with preliminary themes identified and modified, key quotes isolated to illustrate the selected themes, and differences resolved through discussion. The final thematic narrative was then prepared.

## 3. Results

### 3.1. Readiness Survey

Eleven clinician participants provided their retrospective ratings on readiness survey indicators as they pertained to three separate timepoints: (1) pre-COVID-19, (2) early COVID-19, and (3) post-COVID-19 (post-transition to digital delivery). Five of eleven clinician participants had provided digitally delivered care pre-COVID-19, while all eleven clinician participants provided digitally delivered care during early COVID-19 and post-COVID-19.

**Figure 1:**
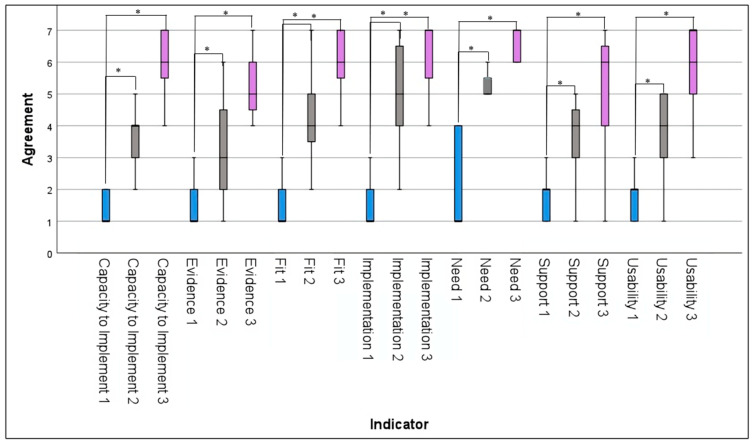
Box plot of median readiness survey indicator scores. Figure 1 is a box plot with an interquartile range showing changes in median readiness survey indicator scores between the pre-COVID-19, early COVID-19, and post-COVID-19 timepoints. 1 = pre-COVID-19 (blue); 2 = early COVID-19 (grey); 3 = post-COVID-19 (purple); * = significant difference based on Wilcoxon signed rank test (*p* < 0.05).

There were significant differences in median scores for all indicators (implementation, need, evidence, fit, usability, capability to implement, and supports) in all pairwise comparisons between the three timepoints as indicated by Friedman’s test (Table 1). The Wilcoxon signed rank test showed significant differences in indicator scores between pairs of timepoints, with scores increasing from timepoint 1 to timepoint 3 (see Figure 1). All 21 statistical tests survived Benjamini–Hochberg FDR correction (see Table 2). In sum, clinician scores increased for all readiness survey indicators across all three timepoints.

### 3.2. Perspectives on Economic Impact

A brief analysis found that 6 out of 11 client participants experienced cost savings using digitally delivered services, saving an average of USD 43 ± 77 per session. Only two out of eleven clinician participants experienced cost savings, with these two participants saving an average of USD 75 ± 49 per session. Ten out of eleven client participants reported saving time using digitally delivered services, saving an average of 98 ± 137 min per session. Similarly, all 11 clinician participants reported time savings using digital services, saving an average of 53 ± 38 min per session.

### 3.3. Interview and Focus Group Results

Descriptive analysis of interview/focus group transcripts with partner participants isolated six main themes: (1) access to care; (2) social conditions; (3) considerations for clinicians; (4) considerations for clients; (5) capacity building; and (6) recommendations for digital mental health delivery. The recommendations made by partner participants are summarized in Table 2.

#### 3.3.1. Theme 1: Access to Care

Community partners generally agreed that DMHI greatly expanded the reach of mental health service provision, allowing clinicians to work with clients trans-provincially in Canada with appropriate licensing. They also noted that digital delivery enabled an increased capacity of care (e.g., an increase in support groups being offered, increasing access to care for clients in more locations) and reduced wait times for services. Increased treatment accessibility led to observed improvement in treatment adherence, overcoming typical barriers to accessing mental healthcare (e.g., travel time, parking, childcare issues). The increased efficiency of clinical resource use was one of the major strengths of digital delivery for community partners. As one participant shared,

*[People] recognize it’s much more pleasant for both the health care provider and the patient to not have to travel and, you know, be physically in a room when they don’t have to be physically in a room. And in particular, you know, even things like, you know, the cost of travel, time, wasted parking, transportation, and even the exposure to an infectious disease in a waiting room, you know, all of that just shouldn’t be things that people are exposed to if it’s not necessary [community partner participant 24].*

#### 3.3.2. Theme 2: Concerns Regarding Treatment Equity

A major concern shared by community partner participants was that social and economic inequalities between individuals might prevent equitable access to digitally delivered care and potentially create greater areas of disparity. Those from lower socioeconomic backgrounds or those living in rural areas may struggle to obtain adequate technological resources, knowledge, and support or access to quiet and private spaces for therapy sessions. Addressing the unique situations and challenges facing such clientele is a clear priority for community partners. As one clinician indicated,

*[The] hiccups tend to be around clients, [what] they own, you know, it’s like technology, […] a computer that’s old, slow internet speeds, microphones that don’t work well, cameras that don’t work well or being out on an acreage and having poor Internet. […] It’s just one of those things that, you know, not much you can do about it, but hopefully there is a solution down the road [community partner participant 17].*

#### 3.3.3. Theme 3: Impact of Digital Delivery on Clinicians 

Community partner participants noted that many clinicians they worked with had experienced frustration and burnout while providing digitally delivered care over the course of the pandemic. The most glaring issue was decreased administrative staff support due to administrative budget cuts. This meant that clinicians were tasked with administrative responsibilities in addition to their clinical caseload, leaving less time between meetings and fewer opportunities to connect with colleagues or take breaks, resulting in increased fatigue and stress. Clinicians also became more available for clients/patients to contact outside of dedicated therapy times due to the normalization of electronic communication (e.g., providing patients with email/phone contact in the event of an emergency). Although beneficial for clients/patients, many clinicians felt that this led to fatigue related to being on Zoom, the phone, or email for longer periods of time. Moving forward, understanding how to balance clinicians’ workloads is a priority for community partners. One partner, for example, described how,

*[W]e had significantly diminished [support] since we transitioned [to digital delivery], which meant a couple of things. Administrative staff was much more difficult to reach and more difficult to [find]. They would seem to be more busy. The sending out Zoom invites and everything else that revolves around setting up meetings and emailing patients and scheduling your own Zoom meetings and connector meetings all separately in two different calendars. It just, it increased the workload for me for administrative needs that were needed to keep my patients significantly supported. There [was no] support [for clinicians]. Support was pulled, if anything [community partner participant 13].*

#### 3.3.4. Theme 4: Impact of Digital Delivery on Clients

A key priority the community partners identified was the need to clarify when and with whom DMHI works best. Community partners shared many characteristics of clients, including their age, severity of their symptoms (e.g., mild vs. severe), and the nature of their home/private environments, that should be considered prior to offering digitally delivered services. The likelihood of client attrition from therapy or isolating themselves should also be investigated more closely. Finally, it was noted that clients should be well educated regarding the risks and benefits of using DHMI prior to starting treatment. Clarifying such information would allow clinicians to have a greater understanding of when and with whom to use such services. As one clinician suggested,

*I see both positive and [negative things in] online support. But as well some limitations [I wouldn’t recommend online support for] children. [And] of course it’s not suitable for elderly people who [have…] It’s problematic for them to use technology, some [may have] cognitive impairment. [And] clients with suicidal ideation, plans, thoughts. So attempts uh, in past and as well. [Those] who are in, I would say, in [an] acute phase of severe mental health disorders, [I would not recommend online support] [community partner participant 7].*

#### 3.3.5. Theme 5: Capacity Building

Community partner group participants, particularly those in managerial positions, found that some clinical spaces lacked the capacity to sustain an increase in clinical load and technological needs long-term. Issues surrounding clinician and client privacy, confidentiality, and security continued to persist in the years following the initial onset of the COVID-19 pandemic. Participants described a need for standardized protocols related to the consenting process and privacy concerns around digital therapy sessions (e.g., is it required to keep cameras on during sessions?). Technological shortages (e.g., lack of telephones and computers for clinicians) and the maintenance of existing technology infrastructure were identified as barriers that must be resolved if DMHI were to be used moving forward. As one manager illustrated,

*[When putting in programs, we need to justify] the business case for it. So what is the need, is [it] exactly what people want? What’s the technology required? What kind of training is required? [What’re] going to be the risks and the challenges, privacy, security, all those kinds of things are sort of big hoops to jump through in order to get others on board and like minded. And so with any kind of change like that, there’s always that sort of resistance, right? So I think that’s the biggest thing we’re putting up against [community partner participant 3].*

Another partner related,

*But then that also came with a whole bunch of other challenges as an organization, we were able to flip very quickly, but not everybody had laptops. So then all of a sudden there was a whole, it wasn’t impossible, but there [were] a few other hurdles that we had to get through because there [were] computer shortages, there were cell phone shortages [community partner participant 2].*

#### 3.3.6. Theme 6: Recommendations for Digital Mental Health Delivery

The majority of community partner participants indicated that DMHI should continue to be used. As such, many of their recommendations focused on how to integrate DMHI into general mental healthcare practice and included: (1) embracing change as an opportunity for system improvement; (2) integrating digital delivery as a standard component of service provision; (3) utilizing technology to support delivery of psychotherapy; (4) ensuring clients have means to receive mental health services digitally; (5) protecting clinician well-being; and (6) aligning policy and practice to support DMH service delivery. A description of each theme and supporting quotes follows.

#### 3.3.7. Sub-Theme 1: Embracing Change as an Opportunity for System Improvement

Contextual factors can present possibilities for enhancing service delivery and catalyzing system change. They also stimulate innovation and use of novel approaches to meet complex population mental health needs. Adopting and supporting these changes is necessary for ensuring the long-term success of DMHI.

*Some people love things to change and love them to change back to the way they were before someone decided to change them. Other people are willing to embrace [the] change. The need is there. It will happen. We’re already further down the road than we thought we’d be. So I’m optimistic. The concept of early adopters. Like they see some change happening and then they’re like, okay, let’s jump on this right now because this is the future sort of deal [community partner participant 23].*

#### 3.3.8. Sub-Theme 2: Integrating Digital Delivery as a Standard Component of Service Provision

There has been significant investment in the infrastructure needed to safely provide DMHI since the onset of the COVID-19 pandemic. Community partners believe digitally delivered services will likely be used for the foreseeable future. Integrating DMHI into general health and mental healthcare practice is the next key step.

*So there’s a lot I mean, if you want to talk about barriers and barriers, like 50 barriers we could talk about at any given time. I think the challenge is, is to start somewhere, is to build a framework so that people can actually get help. We can assess whether that help is working. We reduce the stigma around mental health so people are more prepared to get help [community partner participant 22].*

*In other words, mental health care becomes part of [our] public health care system, which so much of it now is not right. So much of it. It ends up being given to a private insurance or people have to pay out of pocket, which to me is ridiculous [community partner participant 26].*

Community participants felt that addressing clinical concerns specific to digital delivery would aid in future integration of DMHI into general practice.

*How do you address transference in digital therapy delivery? That’s not easy. If it’s about EMDR or technical delivery of therapeutic engagement, that’s relatively easy. But these more analog issues like transference, countertransference are [probably] more difficult, I would guess, to address [community partner participant 3].*

Community participants believed that clients should be allowed the opportunity to choose the mode of delivery they prefer as digital delivery will continue to be commonplace.

*[There’s] always going to be people that prefer the in-person, of course, and mostly what I’m finding, so based on our [clinic] stats as well, is like the older generations are preferring that in-person still, whereas the younger generations are kind of preferring the virtual [community partner participant 14].*

*I’m [a] big advocate for patient choice. I think patients are smart patients. If you give them the right set of facts, they can make educated choices and they should be allowed to make educated choices. So, you know, a lot of these regulatory bodies, what they’re basically saying is we don’t believe patients are smart. We don’t believe we can educate patients, we don’t believe we can trust patients to make the right choices. You properly explain the risks to them, and it cuts across all of these issues. I think a patient, if they say I want, I am okay to have a virtual visit, knowing that that provider may not be able to see me physically, we should allow the patient to take on that choice [community partner participant 22].*

#### 3.3.9. Sub-Theme 3: Utilizing Technology to Support Psychotherapy Delivery

Issues such as treatment suitability and safety and increased administrative burden on clinicians must be addressed. Community partners believe that digital delivery and related innovations can be used to address these issues.

*[I think there are areas Artificial Intelligence could be used], like I tend to think of it from the decision support angle, which is about like, you know, patients identifying sort of key features that then can tell me or tell someone about what might be sort of a risk factor or something that might predict treatment response or a particular illness trajectory. But then I think it also is going to have a big impact in those things like note taking and other sort of menial admin administration tasks. I think, you know, as we sort of talked about, you know, if like time is a big factor for practitioners and I think [Artificial Intelligence] will be useful tool to hopefully reduce some of the burden of certain tasks administratively, whether it be note taking or those types of things follow up [community partner participant 21].*

#### 3.3.10. Sub-Theme 4: Ensuring Clients Have Means to Receive Mental Health Services Digitally

Community partners recognize that not all clients have access to sufficient technological resources to attend digitally delivered sessions. Addressing the potentially non-equitable access to technological resources should be a priority moving forward.

*So I have noticed that some people only have a phone, and so if it’s a phone based connection, it’s sometimes poor quality, then the high speed internet based connections and also depends how many people are on the wifi in the home. It’s surprising how often this happens that people have kids that are on video games and things and streaming, streaming movies and things like that that drops their Internet connection down. So sometimes the quality of the Internet is a problem. Sometimes the quality of the computer is a problem. Some people I mean, you can technically do this work on an iPad or do other dual attention tasks, but it is more challenging [community partner participant 16].*

#### 3.3.11. Sub-Theme 5: Protecting Clinician Well-Being

Clinicians have taken on increased administrative tasks in addition to clinical work, leading to burnout and fatigue from being online for long periods. There is a need for programs to prioritize clinician well-being if DMHI are going to be utilized long-term.

*But it’s also important to look at what impact it had on the mental health of people providing the virtual care. I mean, it’s quite easy to stack a bunch of Zoom meetings back to back to back without any breaks or time to go get a coffee, for example. And then, of course, staring up a screen with blue light all day. That’s hard on the eyes. We’ve heard from some individuals saying, well, we’ve interviewed so far that our clinicians had more headaches because, you know, they’re not moving, they’re not getting outside that idea of social snacking, you know, getting those tidbits throughout your day to just break things up a little bit. It’s quite a bit different than sitting in your kitchen by yourself drinking a cup of coffee for 10 min [community partner participant 15].*

#### 3.3.12. Sub-Theme 6: Aligning Policy and Practice to Support DMH Service Delivery

Community partners identified a need for clarity from regulatory bodies regarding policies and procedures specific to DMHI (e.g., when providing inter-provincial care, which jurisdiction’s policies apply). This would allow for standardization of policies and procedures (e.g., safety protocols) and easier implementation of programs in a wider range of settings.

*And so [if you’re a case manager in Regina for] your client who happens to live in Slave Lake, [and all of a sudden the client] is talking about suicidal ideation, who do [you] call? Like it’s not a 911 call from Regina to respond to Slave Lake. [The] systems have to be in place so that not just the clients are protected, but the clinician, because you’re left holding the bag at the end of a session, or if a client leaves the session and then doesn’t get back to you, how do you follow up? [So yeah], I don’t know that we have the systems actually in place to be able to do much of that cross-border other than in areas where that service has already been happening, like telehealth has been happening for Northwest Territories for a long time [community partner participant 9].*

## 4. Discussion

This study provides insights into the perspectives of clients, clinicians, and community partner participants regarding the current implementation of DMHI, including digitally delivered psychotherapies utilized for trauma-affected populations. Study participants shared that although DMHI have been widely implemented since the onset of the COVID-19 pandemic, many challenges must be addressed as DMHI are further developed. 

The implementation of DMHI changed drastically over the course of the pandemic. Mental health clinicians generally agreed that there were substantial improvements in the fit, feasibility, and overall implementation of DMHI between the “pre-COVID-19” and “post-transition” periods. Generally low agreement scores during the pre-COVID-19 period may be partially attributed to lack of experience using DMHI, as five of our clinician participants had not used such interventions prior to the pandemic. It appears that the need to provide care to clients was one of the main priorities that pushed the implementation of DMHI forward, as indicated by relatively high agreement scores for the Need indicator at the “during transition” and “post-transition” periods. “Post-transition” period scores appear to indicate that clinician participants agreed that DMHI were well implemented, easy to use, and fit well with the needs of their clients. Clinicians also felt that sufficient support was available to access in the event that assistance was needed and agreed that there was a strong evidence base supporting the use of DMHI, which may have influenced their willingness to continue using DMHI in the future. Ultimately, it appears that the success of implementing DMHI at the micro (clinician–patient interactions) and meso (clinic interactions) levels is heavily impacted by how well the interventions meet the needs of clients and clinicians, the ease of use of the interventions, the supports available to clients and clinicians, and the strength of the evidence base supporting such interventions. It is possible that these identified indicators are key to the long-term sustainability and spread of DMHI. Further research should be conducted to verify our results and better understand what readiness survey indicators may impact the implementation of specific interventions, including digitally delivered TFP.

A major benefit of continuing to use DMHI as COVID-19-related restrictions ease is the potential for digital delivery to increase equitable treatment accessibility. Client and clinician participants shared that using DMHI allowed them to more easily and readily access mental health services and save money and time (e.g., savings associated with decreased travel, transportation, parking, and childcare needs), with DMHI being more accessible for clients compared to in-person interventions. This was particularly notable for service delivery to clients residing in rural and remote contexts. Digital delivery may also allow for more efficient use of resources when compared to in-person delivery. Our findings corroborate the results of a previous systematic review, which found that the use of digital health interventions typically leads to increased resource efficiency (e.g., cost savings, increased Quality Adjusted Life Years) for patients [36]. The review further suggests that digital interventions allow for the optimization of available human and technological resources and provide a consistent reduction in healthcare provision costs [36]. The affordability and scalability of DMHI likely will play a major part in reaching high volumes of patients in remote, underserved, or dangerous environments [37]. Better access to mental health treatments may allow for further economic gains, stemming from more disability-free lifespans, lower long-term healthcare expenditure, and higher labor participation [36]. Such economic gains may serve as a funding source for quality improvement, innovation investment, and other regional healthcare needs [37]. The potential economic benefits of DMHI should not be overlooked as these interventions continue to be developed and integrated into mental health treatment practices. An analysis of and further research into cost savings, resource utilization, and efficiency associated with the use of DMHI is warranted. 

Limitations of digital delivery are noteworthy despite the numerous advantages they provide. Community partners cautioned that the increased accessibility of treatment may not necessarily lead to more equitable access to care for clients. Inequitable access to treatment, a longstanding issue in healthcare, can be traced to a variety of potential causes, such as a lack of linguistic capacity, a lack of information regarding where and how to obtain care, and logistical, psychological, and economic barriers (e.g., transportation, childcare, stigma-related concerns, concerns about privacy, long waiting times for services, high costs, or inflexible work schedules) [38]. For digital delivery, difficulties with accessing sufficient technological resources and varying levels of technological knowledge and expertise are disparities that may affect a client’s ability to utilize DMHI. The increased utilization of technological advances, including artificial intelligence, chatbots, and real-time translation, were recommendations made by community partners that may aid in overcoming these barriers within the healthcare system and lead to the increased scale of DMHI. While reporting on specific technologies increasingly being used in digital mental health service delivery was beyond the scope of this study, we acknowledge that the rapidly evolving technological landscape will continue to impact mental healthcare, system processes, and service delivery. Close attention to this evolution and related research is warranted.

The impact of using DMHI on clinicians was another concern raised by community partners. The increase in clinical and administrative responsibilities experienced by some clinicians left them feeling increased tiredness, stress, and burnout following the shift to digital delivery. While clinician participants noted time savings while delivering DMHI, time saved was likely allocated to completing other tasks, such as scheduling client appointments or conducting follow-up appointments, resulting in an increased workload. Less time was also available between online activities for service providers to reflect and consult on cases and practice individually or collectively. Community partner participants also identified difficulties in shifting from providing in-person treatments to DMHI and uncertainty regarding population fit for DMHIs as factors that may have contributed to increased stress. Similar experiences of clinician burnout potentially stemming from factors such as increased screen time, uncertainty regarding the effectiveness of digitally delivered programs, and a loss of access to coping strategies have been detailed in the literature [39,40]. Balancing the needs of clients and clinicians such that clients are receiving effective care without heavily taxing clinicians will aid in ensuring the long-term use and viability of DMHI.

The long-term use and viability of DMHI will also be contingent on improving the micro- (client and clinician), meso (within clinic), and macro-level (government) interactions that arise when providing such services. For example, identifying the most appropriate clientele for DMHI was one area of digital delivery that community partners believed could be improved upon. One potential solution is the increased use of artificial intelligence. These programs could be used to better understand the needs of potential clients and identify the most appropriate treatments for specific diagnostic and demographic characteristics. Further, increasing the availability of educational materials to clients may allow them to better understand the available treatment options and how these treatments can be delivered. These innovations may also lessen the administrative load faced by clinicians, which in turn may allow for greater focus on their clinical caseload. It was also acknowledged that clarity regarding best practices and treatment policies regarding DMHI from regulatory bodies and healthcare authorities (macro-level) was and continues to be necessary to ensure clinicians are comfortable providing digitally delivered care. The integration of clinical research findings, such as trials evaluating the effects of healthcare policy on service provision, may aid in the development of new policies and best practice guidelines. Such interactions may provide a platform for increased collaboration with experts, potentially shortening the knowledge translation cycle. Identifying and addressing the highlighted issues and concerns will be crucial as DMHI continues to be used and integrated into regular clinical practice.

It is clear that the shift to using DMHI following the onset of the COVID-19 pandemic has greatly affected the provision of mental health services. Digital delivery provides many advantages over in-person care, yet many areas of concern remain to be addressed. Building on lessons learned and being intentional, systematic, and explicit around further implementation will support effective and sustainable spread and scale of DMHI. Further investigation in this area is warranted to better understand how to address the issues identified in this study and in previous research.

### Strengths and Limitations

This study captured the distinctive experiences of a highly diverse population, which allowed us to present multiple unique perspectives regarding the current and future implementation of DMHI. There appears to be agreement between all study groups that DMHI has and will continue to provide safe, effective, and accessible care for individuals needing psychotherapeutic treatment. These study results contribute to the evolving literature regarding the use of DMHI. Embracing and integrating digital delivery as a standard component of mental health service provision can lead to significant system improvements, positively impacting clients, clinicians, and the system at large. Use of technology to support the delivery of psychotherapy can enhance client access to needed mental health services. Appropriate clinician training and support, however, is needed to balance caseloads and facilitate their well-being. Finally, aligning policy and practice is essential to ensuring safe and effective DMH service delivery.

Several study limitations must be acknowledged. First, all recruitment, surveys, and interviews in the current study were conducted in English, limiting responses from non-English-speaking communities. Further, the majority of participants were Canadian, which limits the generalizability of study findings, particularly to countries with fewer public mental health treatment options. Additionally, the vast geographic and culturally diverse nature of Canada may lend itself to increased support for using and establishing systems of digital healthcare delivery, potentially limiting the generalizability of study findings to other regions. Although the perspective presented in this study provides crucial insights into the implementation and use of DMHI, given the incredible stress the COVID-19 pandemic placed on public healthcare systems worldwide, further research in other international regions is needed to understand the applicability of DMHI in specific locations. 

Second, the sample size for this study was relatively limited, which prevented us from exploring certain mediating effects, such as gender and sex differences. Recruitment was more challenging than anticipated due to numerous factors, including workplace demands/priorities, research burden on the participant populations, and COVID-19 fatigue. Broadening our recruitment area from Alberta to across Canada and internationally yielded very limited responses. Some clinics that participated in this study saw service delivery changes (e.g., no longer or minimally serving military members, veterans, and public safety personnel, provision of group vs. individual treatment), reducing the number of clinics participating in this study. As a result of these limitations, data collection was concluded prior to reaching our initially planned sample size. A larger sample would have diversified our sample and enabled us to better identify and analyze differing perspectives from the micro, meso, and macro level regarding the use of DMHI. For example, many study participants had successfully used DMHI prior to study participation, potentially biasing their responses. Further, all client participants were military members, veterans, and public safety personnel only, while there was very limited international representation in the community partner group. Research using a more diverse population is needed to clarify the state of implementation of DMHI in various locations and identify the needs and priorities in these areas.

Finally, study participant responses may have been unintentionally biased in favor of supporting DMHI. This study was conducted while COVID-19-related restrictions were commonplace. During this time, digital delivery was one of the few available delivery modalities for receiving and providing mental healthcare. It is possible that this unique context may have unintentionally influenced study participants to provide responses that were overall positive regarding the necessity and efficacy of digitally delivered psychotherapies. Now that the height of the COVID-19 pandemic has passed, it is possible that the positive perceptions of DMHI shared by study participants may change as in-person options become more readily available. For example, some individuals may return to receiving in-person psychotherapy as such options become available, while others may continue to utilize digitally delivered treatments. Further research is warranted to identify client preferences regarding the modality of psychotherapy delivery, as these preferences may greatly influence the types of services offered by mental health treatment teams.

## 5. Conclusions

Client, mental health clinician, and community partner participants generally agreed that DMHI were currently well implemented, with community partner participants believing that such interventions would continue to be used moving forward. As the implementation of DMHI continues, several key issues, such as equitable access to care, mental health clinician well-being, and clarity regarding policies and best practices for DMHI, must be addressed. Being intentional, systematic, and explicit around further implementation will support the effective and sustainable spread and scale of DMHI, maintenance and improvements of the quality of treatment, and leveraging of new developments. Our results may aid in the development and evaluation of DMHI and their related policies and inform future initiatives and research in the field. 

## Figures and Tables

**Table 1:** Results of Readiness Survey Pairwise Comparisons. Summary of Friedman’s tests comparing clinicians’ (*n* = 11) seven readiness survey indicator scores between pairs of timepoints. Chi-square scores were computed using Friedman’s test.

Indicator	Degrees of Freedom	Chi-Square	*p*-Value
Implementation	2	20.829	<0.001
Need	2	19.619	<0.001
Evidence	2	19.158	<0.001
Fit	2	21.535	<0.001
Usability	2	19.158	<0.001
Capacity to Implement	2	21.535	<0.001
Supports	2	17.45	<0.001

**Table 2:** Results of readiness survey post hoc timepoint comparisons. Summary of the Wilcoxon signed rank test comparing clinicians’ (*n* = 11) seven readiness survey indicator scores between pairs of timepoints.

Indicator	z-Score	*p*-Value	Effect Size
Implementation			
Time 2 vs. Time 1	−2.944	0.003 *	0.89
Time 3 vs. Time 1	−2.956	0.003 *	0.89
Time 3 vs. Time 2	−2.565	0.010 *	0.77
Need			
Time 2 vs. Time 1	−2.956	0.003 *	0.89
Time 3 vs. Time 1	−2.958	0.003 *	0.89
Time 3 vs. Time 2	−2.412	0.016 *	0.73
Evidence			
Time 2 vs. Time 1	−2.414	0.016 *	0.73
Time 3 vs. Time 1	−2.944	0.003 *	0.89
Time 3 vs. Time 2	−2.682	0.007 *	0.81
Fit			
Time 2 vs. Time 1	−2.958	0.003 *	0.89
Time 3 vs. Time 1	−2.966	0.003 *	0.89
Time 3 vs. Time 2	−2.877	0.004 *	0.87
Usability			
Time 2 vs. Time 1	−2.546	0.011 *	0.77
Time 3 vs. Time 1	−2.825	0.005 *	0.85
Time 3 vs. Time 2	−2.958	0.003 *	0.89
Capability to Implement			
Time 2 vs. Time 1	−2.848	0.004 *	0.86
Time 3 vs. Time 1	−2.953	0.003 *	0.89
Time 3 vs. Time 2	−2.969	0.003 *	0.90
Supports			
Time 2 vs. Time 1	−2.877	0.004 *	0.87
Time 3 vs. Time 1	−2.825	0.005 *	0.85
Time 3 vs. Time 2	−2.521	0.012 *	0.76

Time 1 = pre-COVID-19; Time 2 = early COVID-19; Time 3 = post-COVID-19. z-scores were computed using post hoc Wilcoxon signed rank tests for pairs of timepoints. * = significant difference that survived Benjamini–Hochberg FDR correction (*p* < 0.05). Effect size based on the following formula: r = *Z*/√N.

## Data Availability

The original contributions presented in this study are included in the article; further inquiries can be directed to the corresponding author.

## Appendix A

Readiness Survey: Digital Delivery of Trauma Therapy

COVID-19 has promoted a change in the way that mental health (MH) services are delivered. While in-person service is often the gold standard, many MH clinicians have been asked to shift to remote delivery of therapies for trauma-affected populations (TAPs) in order to offer timely access so they can continue serving their communities. Through this study, HiMARC will explore whether current digital health trauma therapies work as well as in-person therapy and the impact digital health has on both MH clinicians and TAPs. There is also a need to evaluate the evidence to guide policy, practice, privacy/security, and implementation of digital health. Based on study findings, the HiMARC team will provide recommendations regarding digital health use to inform future decision-making.

The Hexagon Discussion and Analysis Tool is used to evaluate the fit and feasibility of implementing programs or practices in a given context. In this context, we are asking for feedback regarding the implementation of the digital delivery of trauma therapy due to the COVID-19 pandemic.

Implementation

Please indicate the degree to which the digital delivery of trauma therapy was/is implemented at your institution for each of the three timepoints listed below. Please use a scale from 1 to 7, where 1 represents not at all implemented and 7 represents fully implemented.

- Pre-COVID-19: 1---2---3---4---5---6---7
- During transition period: 1---2---3---4---5---6---7
- Currently: 1---2---3---4---5---6---7

**Instructions**

Regarding the digital delivery of trauma therapy at your clinic/institution, please indicate a numerical rating for the following six components at each of three timepoints: (1) pre-COVID-19; (2) during the transition period (transition to digital delivery of trauma therapy, necessitated by COVID-19); and (3) currently. Please use a scale of 1 (low) to 7 (high).

**Need: The Following Section Is about the Need for the Digital Delivery of Trauma Therapy**

Need refers to how well the digital delivery of trauma therapy meets the identified needs of patients, clinicians, and organizations.

Rate “Need” at each of the below timepoints on a scale from 1 (low) to 7 (high):

- Pre-COVID-19: 1---2---3---4---5---6---7
- During transition period: 1---2---3---4---5---6---7
- Currently: 1---2---3---4---5---6---7

**Evidence: The Following Section Is about Evidence for the Digital Delivery of Trauma Therapy**

Evidence refers to the strength of scientific and clinical evidence informing the successful digital delivery of trauma therapy and the outcomes (is it worth it?) that might be expected if it is implemented well. This question is asking you to rate the amount of evidence that existed, to your knowledge, in support of digital delivery of trauma therapy at the three timepoints listed below.

Rate “Evidence” at each of the below timepoints on a scale from 1 (low) to 7 (high).

- Pre-COVID-19: 1---2---3---4---5---6---7
- During transition period: 1---2---3---4---5---6---7
- Currently: 1---2---3---4---5---6---7

**Fit: The Following Section Is about the Fit of Digital Delivery of Trauma Therapy**

Fit refers to how well the digital delivery of trauma therapy aligns with initiatives, structures, and supports within clinics/Alberta Health Services, as well as the priorities of patients and clinicians.

Rate “Fit” at each of the below timepoints on a scale from 1 (low) to 7 (high):

- Pre-COVID-19: 1---2---3---4---5---6---7
- During transition period: 1---2---3---4---5---6---7
- Currently: 1---2---3---4---5---6---7

**Usability: The Following Section Is about the Usability of Digital Delivery of Trauma Therapy**

Usability refers to the degree to which clear practices and processes (both clinical and technological protocols) were/are available to facilitate the digital delivery of trauma therapy, as well as their ease of use and replicability.

Rate “Usability” at each of the below timepoints on a scale from 1 (low) to 7 (high):

- Pre-COVID-19: 1---2---3---4---5---6---7
- During transition period: 1---2---3---4---5---6---7
- Currently: 1---2---3---4---5---6---7

**Capacity to Implement: The Following Section Is about the Capacity to Implement the Digital Delivery of Trauma Therapy**

Capacity to Implement refers to the degree of buy-in of clinicians, patients, and administrators to implement digital delivery of trauma therapy and the availability of resources needed to improve and sustain implementation over time.

Rate “Capacity to Implement” at each of the below timepoints on a scale from 1 (low) to 7 (high):

- Pre-COVID-19: 1---2---3---4---5---6---7
- During transition period: 1---2---3---4---5---6---7
- Currently: 1---2---3---4---5---6---7

**Supports: The Following Section Is about Supports for the Digital Delivery of Trauma Therapy**

Support refers to the extent to which resources (expert assistance, training, staffing, technological assistance, data systems, and administration) were/are available for digital delivery of trauma therapy.

Rate “Supports” at each of the below timepoints on a scale from 1 (low) to 7 (high):

- Pre-COVID-19: 1---2---3---4---5---6---7
- During transition period: 1---2---3---4---5---6---7
- Currently: 1---2---3---4---5---6---7

**Please Offer Any Other Comments:**

## Appendix B

Focus Group Questions

Digital Health Community Partners

COVID-19 Physical Distancing, Virtual Delivery of Trauma Therapies to Trauma-Affected Populations

This interview/focus group follows an iterative process (additional questions may be asked based on the answers to the questions within this guide).

Thank you very much for joining us today. My name is___, and I am a member of the research study entitled “COVID-19 Physical Distancing, Virtual Delivery of Trauma Therapies to Trauma-Affected Populations”, in which you had previously agreed to participate. The intention of bringing you here today is to talk about your experiences, perspectives, thoughts, and feelings around the virtual delivery of trauma therapies for trauma-affected populations.

Before we begin, it is important to ensure you understand your rights and responsibilities in relation to the confidentiality agreement that you signed prior to attending this interview. I will take a moment to review them with you before we begin.

- HiMARC team members (researchers and volunteers) have an ethical responsibility to protect your right to privacy.
- This focus group is being recorded for research purposes.
- To maintain your anonymity, you may choose to leave your camera off or to call yourself by a different name.
- You have the right and responsibility to protect your own privacy and share only what you are comfortable sharing.
- Your choice to say “no”, “yes”, “pass”, “I do not know”, and/or “goodbye” at any time will be respected.
- You have the responsibility to maintain the privacy of focus group participants.
- The HiMARC research team cannot guarantee that focus group participants will maintain the confidentiality of what is discussed.
- You are free to leave the focus group and/or study at any time.

If you have any questions about this study or your rights and/or responsibilities as a participant, we welcome them now, during, and/or after the focus group/interview.

1. There has been a lot of talk about what digital health could be. Is it there? What are the barriers to how it could be deployed?
  If time permits:
  1b. What has worked?
  1c. What has not worked?
  1d. There seems to be a way in which DH can be a part of MH care—what does that say about how we deliver care? Just because we can deliver care, should we? Does DD change how we deliver MH care? There is a relational component to therapy—do we still obtain that when we deliver therapy digitally? In what ways does it change?
2. Can you tell us about your role within the organization?
3. Has your organization been able to implement the digital delivery of trauma therapies?

If so, what has worked well with regard to the virtual delivery of trauma therapies? Prompts: specific barriers (resources and systems).

If not, what have been some of the challenges or barriers with implementing the digital delivery of trauma therapies? Prompts: specific barriers (resources, systems).

4. (if facing significant challenges for implementation) What is needed to implement digitally delivered trauma therapy? (if digital delivery has been implemented widely) What could be improved moving forward?
5. What are some of the advantages/disadvantages to the use of digitally delivered trauma therapies? Prompt: accessibility, safety, fit, effectiveness, etc.

Thank you for joining us today. Know that we value your willingness to share your experience with us.

Before we conclude our focus group, we would like to take a moment to review the confidentiality rights and responsibilities:

- HiMARC team members (researchers and volunteers) have an ongoing ethical responsibility to protect your right to privacy.
- This focus group was recorded for research purposes.
- You have an ongoing responsibility to maintain the privacy of focus group participants.
- The HiMARC research team cannot guarantee that focus group participants will maintain the confidentiality of what has been discussed.
- You are free to withdraw your focus group and/or study data at any time.

If you have any questions about this study or your rights and/or responsibilities as a participant, we welcome them now and/or in the future.
